# Editorial Notes

**Published:** 1882-09

**Authors:** 


### EDITORIAL NOTES.

Our printer inadvertently failed to credit the very practical article on the "Treatment of Acne" in the selection of the August number to the *St. Louis Medical and Surgical Journal*, from which it was taken. Our apologies are due to the editor of the above journal for this omission.

THE *London Lancet*, after noticing the recent action of the American Medical Association in refusiug admission to the delegates of the Medical Society of the State of New York, says: "When will the British Medical Association take a similar course and refuse membership to those who express their willingness to consult with homœopathic and other irregular, though qualified, practitioners?"

AT the Jubilee meeting of the British Medical Association "The Report of the Council" states, that, as far as possible, the Association had closed the door of entrance to a professing homœopath. They considered it unadvisable to take action for the expulsion of any who had perverted to homœopathy after admission, because 1st, Such a course would be beneath the dignity of the members of a great and avowedly liberal profession, and 2d, Because it would confer an amount of notoriety which was very undesirable upon those which were expelled.

